# Antiviral Activity of Crude Polysaccharide Derived from Seaweed against IHNV and IPNV In Vitro

**DOI:** 10.3390/v14092080

**Published:** 2022-09-19

**Authors:** Guangming Ren, Liming Xu, Jingzhuang Zhao, Yizhi Shao, Yujie Lin, Linfang Li, Qi Liu, Tongyan Lu, Qiya Zhang

**Affiliations:** 1 Key Laboratory of Aquatic Animal Diseases and Immune Technology of Heilongjiang Province, Department of Aquatic Animal Diseases and Control, Heilongjiang River Fisheries Research Institute, Chinese Academy of Fishery Sciences, Harbin 150070, China; 2 State Key Laboratory of Freshwater Ecology and Biotechnology, Institute of Hydrobiology, Chinese Academy of Sciences, Wuhan 430072, China

**Keywords:** IHNV, IPNV, co-infection, antivirus, polysaccharide

## Abstract

Both infectious hematopoietic necrosis virus (IHNV) and infectious pancreatic necrosis virus (IPNV) are the causative agents of acute and highly contagious diseases of juvenile salmonids, resulting in severe economic losses to these cold-water fish globally. There is an urgent need to explore antiviral agents against IHNV and IPNV due to the lack of commercially available vaccines and antiviral drugs. More importantly, the co-infection of IHNV and IPNV is prevalent in nature, which not only aggravates extensive damage to the salmonids but also poses challenges to its prevention and control. The antiviral effects of a crude polysaccharide derived from seaweed (CSP) on IHNV and IPNV were evaluated in this study separately. Furthermore, the underlying antiviral mechanisms of CSP to IHNV and IPNV were analyzed, respectively. The results showed that CSP possessed excellent safety and good ability to inhibit IHNV, IPNV, and their co-infection. CSP preferred to act at the early stage of viral infection. The antiviral mechanism of CSP on IHNV is possibly involved in preventing viral attachment and release, while in IPNV, it is involved in suppressing viral attachment, entry, and release. Taken together, the results of this study shed new light on developing novel agents against viral infection in salmonid fish.

## 1. Introduction

Infectious hematopoietic necrosis virus (IHNV) and infectious pancreatic necrosis virus (IPNV) are the critical pathogens that cause high salmonid mortality worldwide. IHNV belongs to the genus *Novirhabdovirus* within the family Rhabdoviridae and is a negative-strand RNA virus with a genome of approximately 11 kb that encodes six proteins: nucleocapsid (N), polymerase-associated phosphoprotein (P or M1), a matrix protein (M or M2), a surface glycoprotein (G), a non-virion protein (NV), and a virus RNA polymerase (L) [1]. It is listed in the Aquatic Animal Health Code released by the Office International des epizooties (OIE) [2]. IPNV belongs to the genus *Aquabirnavirus* within the family Birnaviridae and contains two double-stranded RNA segments (A, B). Segment A encodes the proteins VP2, VP4, and VP3, and segment B encodes L protein VP1 [3]. Since their first discoveries during the 1950s, IHNV and IPNV have become endemic and rapidly spread to the many salmonid hatcheries of America, Europe and Asia [4,5,6,7]. Both IHNV and IPNV infection can cause 90% cumulative mortality or higher in acute outbreaks depending on the fish species and size, viral strain, and environmental conditions [8,9].

It is well known that IHNV and IPNV can induce a virus-carrier state in surviving fish and establish persistent infection, resulting in a highly contagious and destructive disease [3,10]. This persistence of viruses in fish easily causes the natural co-infection of IHNV and IPNV. Co-infection of IHNV and IPNV has been found in many salmonid hatcheries and farms in the US, some of Europe, and Asia [8,9,10,11,12,13]. This not only brings extensive damage to the salmonid industry but also poses a huge challenge to prevention and control. It is preferable to prevent viral infections than to treat them. There are commercial vaccines for fish to prevent IHNV and IPNV, but the applications of these vaccines are limited in most countries due to the differences in viral serotypes and security-related factors, etc. [9,14]. Considering the long and complicated process of vaccine development, the exploration of natural antiviral compounds has attracted increasing attention. Polysaccharides, as natural immunostimulants, can directly or indirectly enhance the immune functions of the host and decrease the risk of diseases [15]. Moreover, they have the advantages of a wide range of sources, high biological activity, low toxicity, and few adverse reactions. Currently, polysaccharides have been successfully used in health food and medicine due to their diverse pharmacological activities, including anti-oxidation, antivirus effects, anti-inflammation effects, and immuno-regulation [16]. Cumulative studies have reported that polysaccharides derived from seaweeds (algae) demonstrate a great capability to inhibit the infection of viruses such as human immunodeficiency virus, herpes simplex virus, dengue virus, coronavirus, influenza A virus, metapneumovirus, vesicular stomatitis virus, white spot syndrome virus, etc. [17,18,19,20,21,22,23,24,25,26]. The polysaccharides exhibit these antiviral effects possibly via inhibiting one or more steps of viral infection, including virus attachment, entry, replication, and release. Moreover, these polysaccharides are reported to strengthen host immune defense functions and, therefore, can be developed as immunostimulants. Polysaccharides could present a potential application prospect for the prevention and control of viral diseases in aquaculture. However, the information underlying the polysaccharides against aquatic viruses like IHNV, IPNV, and their co-infection is still limited. Therefore, it is necessary to exploit the polysaccharides that can be used as a functional ingredient or agent for preventing IHNV, IPNV, and their co-infection.

In this study, the antiviral capabilities of crude polysaccharide derived from seaweed (CSP) to IHNV and IPNV were determined in vitro. The time-of-addition assays were performed to explore the acting stage of CSP in IHNV and IPNV infection, respectively. Moreover, the inhibitory mechanisms of CSP underlying IHNV and IPNV infection were further analyzed in vitro, separately. In addition, the antiviral activity of CSP to the co-infection of IHNV and IPNV was evaluated. The results provide a novel therapeutic agent for preventing salmonids from viral diseases.

## 2. Materials and Methods

### 2.1. Cell and Virus

The Chinook salmon embryo (CHSE-214) cell line was maintained at 18 °C with 5% CO_2_ atmosphere in a complete medium (minimum essential medium growth medium (Hyclone, Logan, UT, USA) supplemented with 10% fetal bovine serum (FBS) (Gibco BRL, Grand Island, NY, USA) and 100 units/mL of penicillin and streptomycin). IHNV-sn1203 and IPNV-ChRtm213 were isolated from diseased rainbow trout and stored in our laboratory as previously described [27,28].

### 2.2. Preparation of CSP

The crude polysaccharide was obtained from the dried *Laminaria japonica* by hot water extraction and ethanol precipitation with a yield of 8.41%. In brief, dried *Laminaria japonica* was grounded and then suspended in the distilled water at 60 °C for 3.0 h. The supernatant was collected using the Buchner funnel and precipitated by adding an equal volume of ethanol to a final concentration (80%, *v*/*v*). The obtained polysaccharide solution was purified with the savage reagent, then dialyzed and lyophilized after concentrating with a rotary evaporator under reduced pressure. CSP powder is soluble in water and insoluble in ethanol, acetone, and ether. The polysaccharide content of CSP was determined by the anthrone-sulfuric acid colorimetric method [29], and the polysaccharide percentage of CSP was 6.53%. CSP was dissolved with a maintenance medium (minimum essential medium growth medium supplemented with 2% FBS, 100 units/mL of penicillin and streptomycin) for the indicated concentration.

### 2.3. Cytotoxicity Assays

CHSE-214 cells with a density of 1 × 10^4^ per well were seeded into a 96-well plate and cultured to reach approximately 90% confluence in each well. Subsequently, cells were exposed to maintenance medium with different concentrations of CSP (100, 200, 300, 400, 500, 1000, 2000 μg/mL). Cells were cultured with a maintenance medium without adding CSP and used as the mock control. The plates were incubated at 15 °C with a 5% CO_2_ atmosphere for 7 days. Cell viability was determined using the 3-(4,5-dimethythiazol-2-yl)-2,5-diphenyltetrazolium bromide (MTT) colorimetric method [30]. The cell viability rate was calculated as per Formula (1).

(1) $$\mathrm{Cell}\;\mathrm{vialibilty}=\frac{{\mathrm{OD}}_{T}-{\mathrm{OD}}_{B}}{{\mathrm{OD}}_{M}-{\mathrm{OD}}_{B}}\times 100\%$$

were OD_T_, OD_M__,_ and OD_B_ referred to the absorbance of the CSP control, mock control, and blank control groups, respectively.

### 2.4. Determination of Antiviral Abilities In Vitro

CHSE-214 cells with a density of 1 × 10^4^ per well were seeded into a 96-well plate and cultured to reach approximately 90% confluence in each well. Cells were infected with IHNV or/and IPNV (MOI, 0.1 and 1.0) in the presence of CSP (100, 200, 300, 400, 500 μg/mL), respectively. Cells without additional treatments were set as the mock control. Cells without adding CSP but infected with the virus were set as the challenge control. Cell viability was detected using MTT colorimetric method when the cytopathic effect (CPE) in the challenge group reached approximately 75%. The antiviral activity of CSP was calculated as per Formula (2).

(2) $$\mathrm{Antiviral}\;\mathrm{activity}=\frac{{\mathrm{OD}}_{T}-{\mathrm{OD}}_{C}-{\mathrm{OD}}_{B}}{{\mathrm{OD}}_{M}-{\mathrm{OD}}_{C}-{\mathrm{OD}}_{B}}\times 100\%$$

where OD_T_, OD_M_, OD_C_, and OD_B_ referred to the absorbance of the CSP control, mock control, challenge control, and blank control groups, respectively.

### 2.5. Time-of-Addition Assay

CHSE-214 cells with a density of 1 × 10^4^ per well were seeded into a 96-well plate and cultured to reach approximately 90% confluence in each well. CHSE-214 cells were infected with IHNV/IPNV at a MOI of 1.0 and then incubated for 1 h at 15 °C with a 5% CO_2_ atmosphere. Indicated concentrations (100, 200, 300, 400, 500 μg/mL) of CSP were administered prior to infection (−12, −6, −1 h) as well as at 1, 2, 6 and 12 h post-infection with the virus. Cell viability was detected using the MTT colorimetric method when the CPE in the challenge group reached approximately 75%. The treatments of other groups and the determination of antiviral activity were referred to in Section 2.4.

### 2.6. Inactivation Assay

CSP (100, 200, 300, 400, 500 μg/mL) were together incubated with IHNV and IPNV (MOI, 1.0) at 15 °C for 1, 2, and 6 h, respectively. CHSE-214 cells with a density of 1 × 10^4^ per well were seeded into a 96-well plate and cultured to reach approximately 90% confluence in each well. Subsequently, the incubated solutions were added to the cells and cultured at 15 °C with a 5% CO_2_ atmosphere. Cell viability was detected using the MTT colorimetric method when the CPE in the challenge group reached approximately 75%.

### 2.7. Inhibitory Action Assays

Regarding viral attachment, cells were challenged with the virus (MOI, 10.0) and 200 μg/mL of CSP under 4 °C and then promptly incubated at 4 °C for 1 h. Regarding viral entry, cells were challenged with the virus (MOI, 10.0) under 4 °C and then incubated at 4 °C for 1 h. Subsequently, the cells were washed thrice with cold PBS, re-overlaid with 200 μg/mL of CSP, and incubated at 15 °C with a 5% CO_2_ atmosphere for 1 h. Cells were washed thrice with citrate buffer (pH, 3.0) and cold PBS, respectively. In viral replication, cells were challenged with the virus (MOI, 10.0) and then incubated at 4 °C for 1 h. Cells were washed thrice with cold PBS and incubated at 15 °C with a 5% CO_2_ atmosphere for 2 h. Cells were washed, re-overlaid with 200 μg/mL of CSP solution, and then incubated at 15 °C with a 5% CO_2_ atmosphere for 4 h. The mRNA levels of IHNV-L/IPNV-VP2 with the above treatments were determined using RT-qPCR. Regarding viral release, cells were challenged with the virus (MOI, 10.0) and incubated at 15 °C with a 5% CO_2_ atmosphere for 12 h. Cells were re-overlaid with 200 μg/mL of CSP solution and then incubated at 15 °C with a 5% CO_2_ atmosphere for 30, 60, and 90 min, respectively. The supernatants were collected to detect the titers (TCID_50_) using the Reed–Muench method.

### 2.8. IFAT

Infected CHSE-214 cell monolayers were processed for IFAT at 48 h post-IHNV inoculation as follows: monolayers were fixed, permeabilized, and incubated with the primary and secondary antibodies. After several washes, monolayers on the 6-well plate were observed by a fluorescence microscope (Leica, Wetzlar, Germany). The secondary antibody labeled with CY3 (red fluorescence) was used to visualize IHNV, and the secondary antibody labeled with FITC (green fluorescence) was used to visualize IPNV.

### 2.9. RT-qPCR Analysis

RNA was extracted using Trizol (Invitrogen, Carlsbad, CA, USA) according to the manufacturer’s instructions. RT-qPCR was performed in triplicate on an ABI Prism 7500 Sequence Detection System (Life Technologies) using the One Step SYBR PrimeScript PLUS RT–PCR Kit (Perfect Real Time) (Takara, Shiga, Japan). Β-actin was used as an internal control for the assay of an expressed gene. Fold changes of each gene expression level were calculated based on 2^−ΔΔCt^. The sequences of primer pairs are shown in Table S1. All reactions were performed in triplicate.

### 2.10. Statistics Analysis

Results were expressed as the mean ± standard deviation (SD), and statistical analysis was performed using a one-way analysis of variance (ANOVA) with a *t*-test (SPSS 19.0, SPSS Inc., Chicago, IL, USA). The criterion of significance was conducted at *p* < 0.05.

## 3. Results

### 3.1. Cytotoxicity of CSP In Vitro

The survival rate of CHSE-214 cells reached 103.97% when the CSP concentration was 100 μg/mL (Figure S1). The cell survival rate was up to 90% when the CSP concentration was 500 μg/mL. Conversely, the cell viability decreased to 76.70% when the CSP concentration increased to 2000 μg/mL. The results showed that CSP was not cytotoxic to CHSE-214 cells to some extent, with a CC_50_ of 4418 μg/mL.

### 3.2. Antiviral Activity of CSP on IHNV

#### 3.2.1. Antiviral Effects of CSP on IHNV

**Figure 1:**
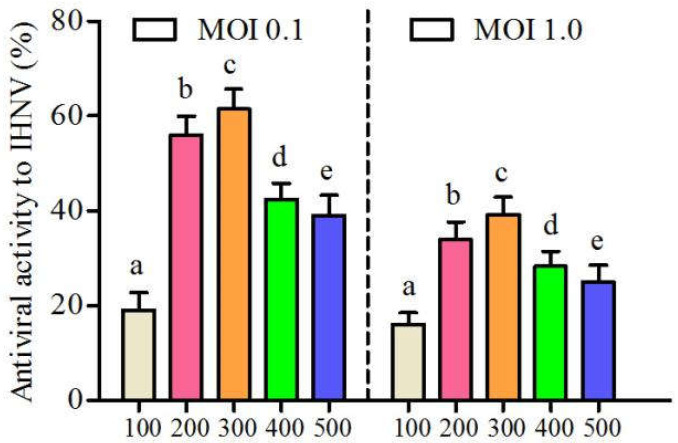
The antiviral activity of CSP to IHNV with a MOI of 0.1 and 1.0, respectively (different letters indicate statistical significance, *p* < 0.05).

CSP exhibited good antiviral effects on IHNV both at a MOI of 0.1 and 1.0 (Figure 1). The results showed that the inhibitory effects of CSP increased with an increasing CSP concentration ranging from 100~300 μg/mL and then gradually declined with the increasing concentration. Antiviral activities reached the peaks of 61.44% and 39.10% at a MOI of 0.1 and 1.0, respectively, when the CSP concentration was 300 μg/mL. The above results indicated that CSP had inhibitory ability against IHNV to some extent.

#### 3.2.2. Determination of Time-Addition Mode of CSP to IHNV

**Figure 2:**
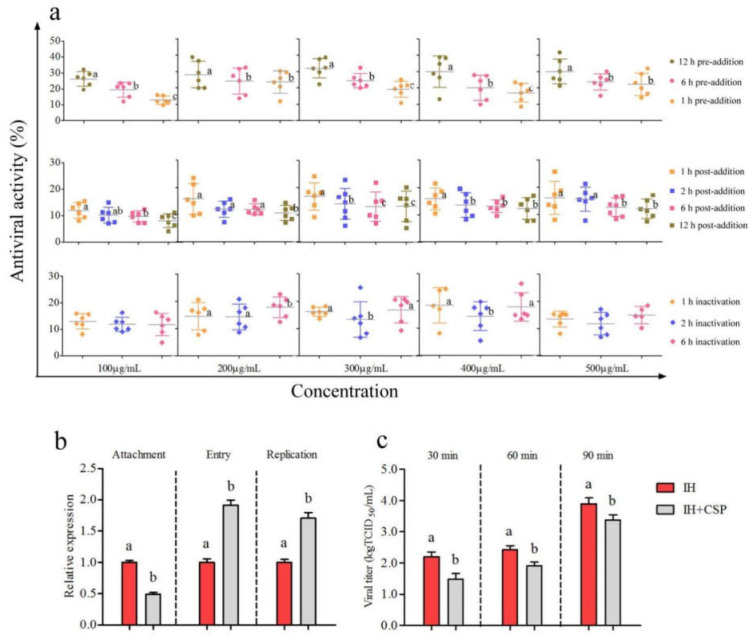
The antiviral activity and actions of CSP on IHNV. (**a**) The antiviral activity of CSP with different concentrations on IHNV under the pre-addition, post-addition, and inactivated treatments. (**b**) The changes of relative expression levels of IHNV-L gene in the viral attachment, entry, and replication processes, respectively. (**c**) The determination of the viral titers during the viral release process. Different letters indicate statistical significance, *p* < 0.05.

CSP exhibited stronger inhibition at the same concentration with 12 h of pre-addition than with 1 h and 6 h of pre-addition (*p* < 0.05) (Figure 2a). The inhibitory activity reached 32.01% with 12 h of pre-addition when the CSP concentration was 300 μg/mL. There were slight inhibitory effects of CSP on IHNV with 1 h, 2 h, 6 h, and 12 h of post-addition (8.17~16.77%) at concentrations ranging from 100 to 500 μg/mL (Figure 2a). However, the inhibitory activities of CSP at the same concentration with 1 h of post-addition were relatively higher than those with 2 h, 6 h, and 12 h of post-addition. Furthermore, CSP had a slightly inactivating effect on IHNV at concentrations ranging from 100 to 500 μg/mL. There was no significant increment in CSP inactivated activity at the same concentration with the prolongation of incubated time (*p* > 0.05). The inactivated activity of 300 μg/mL CSP reached 16.15% with 1 h of co-incubation (Figure 2a). The above results indicated the effects of CSP on inhibiting IHNV with pre-addition were stronger than with post-addition and co-incubation.

#### 3.2.3. Analysis of Inhibitory Action of CSP to IHNV

There was a significant decrease in the mRNA level of IHNV-L after CSP treatment compared with the IH group during the attachment process (*p* < 0.05) (Figure 2b). Unexpectedly, CSP treatment significantly increased the mRNA levels of IHNV-L compared with the IH group during the entry and replication processes (*p* < 0.05). However, IHNV titers significantly declined after CSP incubation of 30 min, 60 min, and 90 min, respectively (Figure 2c). The results indicated that CSP exerted good inhibitory effects on IHNV, possibly through intervening in the viral attachment and release processes.

### 3.3. Antiviral Activity of CSP on IPNV

#### 3.3.1. Antiviral Capabilities of CSP on IPNV

**Figure 3:**
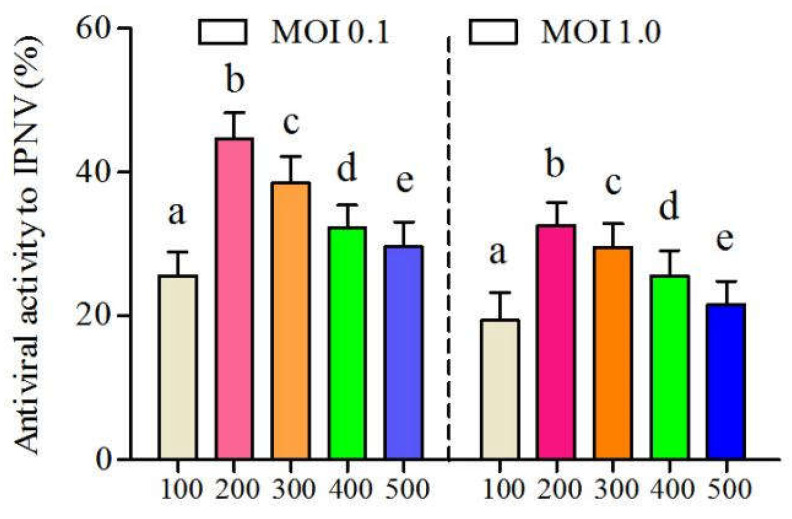
The antiviral activity of CSP on IPNV with a MOI of 0.1 and 1.0, respectively (different letters indicate statistical significance, *p* < 0.05).

CSP exhibited a potential antiviral effect on IPNV (Figure 3). Antiviral activities of CSP on IPNV both at a MOI of 0.1 and 1.0 reached the peaks (44.69 and 32.58%) when the concentration was 200 μg/mL. Subsequently, its inhibitory effects on IPNV gradually decreased with the increase in CSP concentration. Antiviral activities of CSP decreased to 29.63% and 21.59%, respectively, at a MOI of 0.1 and 1.0 when the CSP concentration was 500 μg/mL.

#### 3.3.2. Determination of Time-Addition Mode of CSP on IPNV

**Figure 4:**
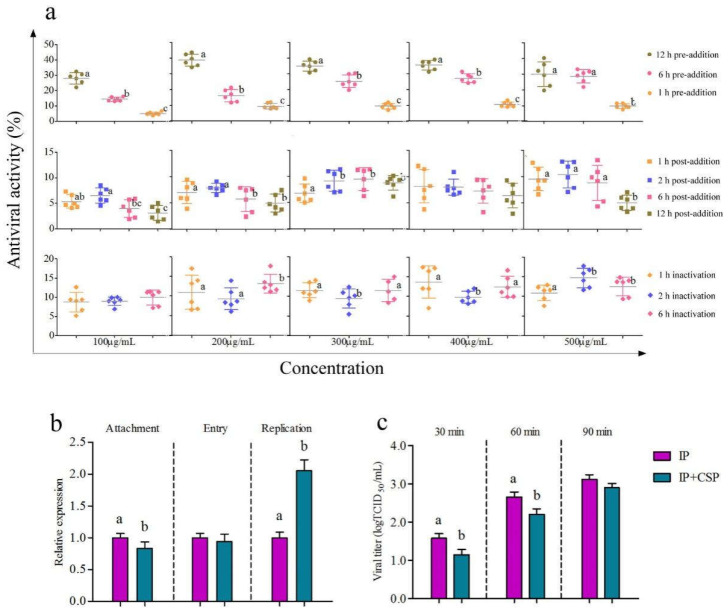
The antiviral activity and actions of CSP on IPNV. (**a**) The antiviral activity of CSP with different concentrations on IPNV under the pre-addition, post-addition, and inactivated treatments. (**b**) The changes in relative expression levels of the IPNV-VP2 gene in the viral attachment, entry, and replication processes, respectively. (**c**) The determination of the viral titers during the viral release process. Different letters indicate statistical significance, *p* < 0.05.

The inhibitory effects of CSP to IPNV with the pre-addition were similar to those of IHNV. Inhibitory effects of CSP to IPNV at the same concentration were significantly elevated with the prolongation of pre-addition time (*p* < 0.05) (Figure 4a). Antiviral activities of CSP with 12 h of pre-addition were all higher than those with 1 h and 6 h pre-addition, respectively. Antiviral activities of CSP on IPNV reached 27.99%, 38.71%, 34.98%, 35.54% and 30.04% with 12 h of pre-addition when CSP concentrations were 100, 200, 300, 400 and 500 μg/mL, respectively. There was a slight inhibitory effect of CSP on IPNV with the post-addition mode (Figure 4a). On the whole, the antiviral effects of CSP declined with the prolongation of IPNV infection. Furthermore, CSP exhibited slightly inactivating effects on IPNV at concentrations ranging from 100 to 500 μg/mL (Figure 4a). Similarly, there was no significant difference in the inactivating effect of CSP on IPNV with the increases in co-incubated time. The above results suggested that the roles of CSP in suppressing IPNV with pre-addition were stronger than those with post-addition and co-incubation.

#### 3.3.3. Analysis of Inhibitory Action of CSP on IPNV

As shown in Figure 4b, CSP treatment decreased IPNV-VP2 mRNA levels during the attachment and entry processes. There were significant increments in the mRNA levels of IPNV-VP2 after CSP treatment in the viral replication process (*p* < 0.05). In addition, CSP could block IPNV release, which was represented by the significant decline in viral titers, especially at 30 and 60 min (*p* < 0.05) (Figure 4c). The above results indicated that the antiviral activity of CSP on IPNV was probably related to the involvement of viral attachment, entry, and release processes.

### 3.4. Antiviral Capability of CSP on Co-Infection of IHNV and IPNV

**Figure 5:**
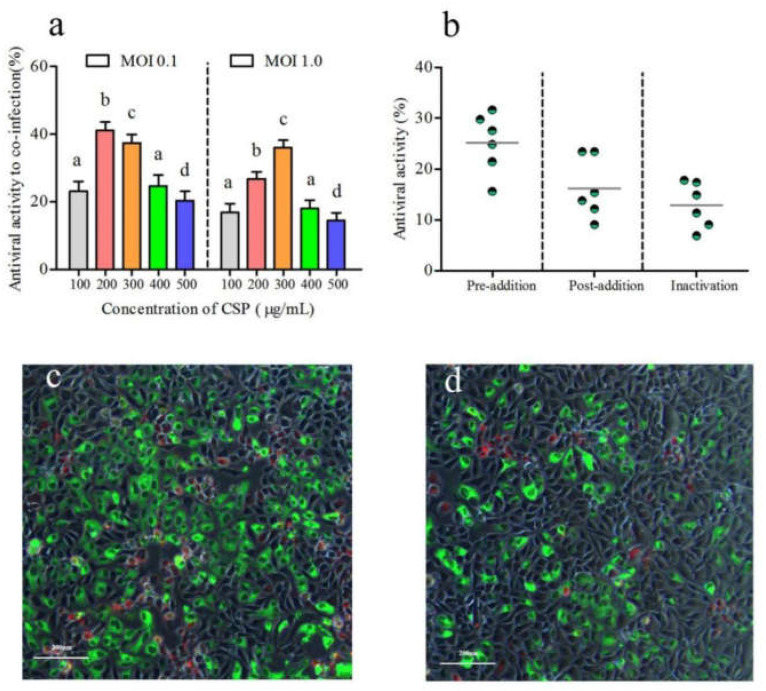
The antiviral ability of CSP on the co-infection of IHNV and IPNV. (**a**) The antiviral activity of CSP on the co-infection of IHNV and IPNV with a MOI of 0.1 and 1.0, respectively. (**b**) The antiviral activity of CSP on IPNV under the pre-addition, post-addition, and inactivated treatments. (**c**) IFAT result of the co-infection without the presence of CSP. (**d**) IFAT result of the co-infection with the presence of CSP. Different letters indicate statistical significance, *p* < 0.05).

The inhibitory activity of CSP on co-infection of IHNV and IPNV at a MOI of 0.1 was up to 41.06% when the CSP concentration was 200 μg/mL (Figure 5a). Its antiviral effect at a MOI of 1.0 reached a peak of 36.02% when the CSP concentration was 300 μg/mL. As shown in Figure 5b, the inhibitory activities of CSP on the co-infection reached 25.16%, 16.24%, and 12.92%, respectively, with 12 h of pre-addition, 1 h of post-addition, and 1 h of inactivation. The results suggested that the antiviral effect of CSP on the co-infection with the pre-addition mode was stronger than the others. In addition, a severe cytopathic effect was observed in the co-infection of IHNV and IPNV with the absence of CSP based on the IFAT analysis (Figure 5c). Conversely, the presence of CSP notably decreased the cytopathic effect, which was presented by decreases in the specific red and green fluorescent signals (Figure 5d). The above results proved that CSP exerted a good ability to inhibit the co-infection of IHNV and IPNV to an extent.

## 4. Discussion

Despite the progress made in vaccine research and development, both IHNV and IPNV are still the causative agents of the most important viral diseases in farmed salmonids worldwide [8,9]. Numerous efforts have been made toward the development of antiviral agents in salmonids due to the lack of commercially available drugs and vaccines to prevent and treat these viral diseases. Polysaccharides as natural immunostimulants possess potential therapeutic properties, including immune modulation, anti-inflammatory, antibacterial and antiviral activities [16,17,18,19,20,21,22,23,24,25,26]. A previous study had shown that polysaccharides exerted antiviral abilities on IHNV [16,31]. However, the information is limited concerning the use of the polysaccharide against IPNV. In the present study, the obtained CSP possessed excellent safety and promising antiviral activity against IHNV and IPNV, as well as their co-infection.

The whole process of viral propagation mainly includes viral attachment, entry, replication, and release. In order to better explore the inhibitory actions of CSP on both IHNV and IPNV, the same concentration of CSP was chosen to perform the inhibitory action assays. The assays where CSP was added to the infected cell monolayers before and after the virus inoculation revealed that CSP preferred to act at the early stage of IHNV and IPNV infection. As we all know, viral attachment to host tissues is the early event of virus infection and is a prerequisite for initiating the majority of infectious diseases [32]. It is widely accepted that the antiviral mechanisms of most polysaccharides mainly involve the inhibition of the adsorption of viruses onto host cell surfaces [33,34]. Generally, polysaccharides exhibit inhibitory activity on viral infection by targeting the attachment phase mainly using two strategies, including the interaction with virions and mimicking the virus-associated protein to bind with the receptors [35,36,37,38]. The formed virion-polysaccharide complex could occupy the viral binding sites required for viral attachment to the host cell and thus render the virus unable to complete the subsequent infectious process [39,40]. The weak inactivated effects of CSP on IHNV indicated that CSP might partially interact with the virus to form the virion-CSP complex, while its significant role in preventing the attachment of IHN virus particles onto the host cell surface showed that CSP targeting the attachment phase could be involved in the competition of the receptors with the virus via the putative receptor-binding domain, preventing viral adsorption to the host cell surface (*p* < 0.05). It was reported polysaccharides derived from *Chlorophyta*, *Ulvaceae*, *Cladosiphon okamuranus*, etc. also exerted good antiviral activities via predominantly suppressing the initial process of virus attachment [19,20,21,22]. Some studies indicated that the antiviral effects of polysaccharides might be achieved by interfering with different steps of the virus life cycle [32,33,34,35,36,37,38]. They exhibited antiviral activities, possibly via inhibiting the attachment of the virus, hindering the entry of the virus into host cells, or preventing the virus’ replication and release. In the present study, there were no inhibitory effects of CSP on IHNV entry, transcription, and replication, but there were effects on release. The results suggested that CSP also played an inhibitory role in keeping the viral particles in the cell not released to the outside to the extent. Similar results were observed in the studies of Wang et al. (2016) and Ming et al. (2017), where the sulfated *Astragalus* polysaccharide and *Chrysanthemum Indicum* polysaccharide could inhibit the infection of duck hepatitis A virus via suppressing the viral release [41,42].

IPNV, different from IHNV, has no receptor- or surface-interacting molecules for its adsorption [43]. However, IPNV can induce fluid-phase capture in a dose-dependent fashion and trigger the appropriate signaling pathway to elicit macropinocytosis capture for its entry. The transient increase in fluid-phase uptake is conducive to macropinocytosis [44]. The suppression of CSP on IPNV attachment and entry indicated that it might influence the fluid-phase uptake in CHSE-214 cells by interacting with the virus to form the virion-CSP complex and subsequently inhibiting the further formation of the macropinosome. Likewise, with IHNV, CSP had no inhibitory effects on IPNV transcription and replication, but there were effects on release. Some researchers reported that polysaccharides as a macromolecule have difficulty in entering into cells [45]. This might explain why there were no effects of CSP on both the IHNV and IPNV replication processes. The results indicated CSP played a significant role in suppressing the release of IPNV from the cells. In addition, previous studies reported that polysaccharides from natural sources possessed antiviral activities via direct virucidal effects [17,46,47,48,49]. In this study, the direct virucidal effect of CSP on viruses was relatively weak. The above results indicated that the inhibitory mechanisms of CSP involved complicated and multiple processes.

As expected, CSP exhibited a good capacity to inhibit the co-infection of IHNV and IPNV. The inhibitory actions of CSP on the co-infection of IHNV and IPNV were similar as those acting on the single infection of IHNV and IPNV, demonstrating a tendency to act at the early stage of co-infection. It is well known that a co-infection is a complicated infectious process due to the complex nature of viral interactions, occurring either via synergistic or antagonistic effects [50,51,52]. Hence, the antiviral mechanisms of these polysaccharides against the co-infection of IHNV and IPNV remain to be further explored. It will be crucial to pursue the application of antiviral agents to prevent and treat infectious viral diseases in aquaculture.

## 5. Conclusions

In conclusion, CSP significantly inhibited IHNV infection by preventing viral attachment and release in the host cells. It also exhibited anti-IPNV activity through suppressing viral attachment, entry, and release. Furthermore, CSP presented a good capacity to inhibit the co-infection of IHNV and IPNV. The results showed that CSP is a promising polysaccharide for developing new inhibitory agents against IHNV, IPNV, and their co-infection.

## Data Availability

All of the materials and data that were used or generated in this study are described and available in the manuscript.

## Supplementary Materials

The following supporting information can be downloaded at: https://www.mdpi.com/article/10.3390/v14092080/s1, Table S1: Primer sequences for RT-qPCR analysis; Figure S1: Cytotoxicity analysis of CSP to CHSE-214 cells.

## References

[1] Kurath G., Garver K.A., Troyer R.M., Emmenegger E.J., Einer-Jensen K., Anderson E.D. (2003). Phylogeography of infectious haematopoietic necrosis virus in North America. J. Gen. Virol..

[2] OIE World Organization for Animal Health, Aquatic Animal Health Code. http://www.oie.int/international-standardsetting/aquatic-code/access-online.

[3] Alonso M., Rodriguez Saint-Jean S., Perez-Prieto S.I. (2003). Virulence of infectious hematopoietic necrosis virus and infectious pancreatic necrosis virus coinfection in rainbow trout (*Oncorhynchus mykiss*) and nucleotide sequence analysis of the IHNV glycoprotein gene. Arch. Virol..

[4] Rucker R.R., Whippie W.J., Parvin J.R. (1953). A contagious disease of salmon possibly of virus origin, US Fish Wildl. Serv. Fish Bull..

[5] Dixon P., Paley R., Alegria-Moran R., Oidtmann B. (2016). Epidemiological characteristics of infectious hematopoietic necrosis virus (IHNV): A review. Vet. Res..

[6] Enzmann P.J., Castric J., Bovo G., Thiery R., Fichtner D., Schütze H., Wahli T. (2010). Evolution of infectious hematopoietic necrosis virus (IHNV), a fish rhabdovirus, in Europe over 20 years: Implications for control. Dis. Aquat. Org..

[7] Wood E.M., Snieszko S.F., Yasutake W.T. (1995). Infectious pancreatic necrosis in brook trout AMA Arch. Pathology.

[8] Bootland L.M., Leong J.C., Woo P.T.K., Bruno D.W. (1999). Infectious hematopoietic necrosis virus. Fish Diseases and Disorders.

[9] Santi E.N., Mahy B.W.J., Van Regenmortel M.H.V. (2008). Infectious pancreatic necrosis virus. Encyclopedia of Virology.

[10] Dadar M., Memari H.R., Vakharia V.N., Peyghan R., Shapouri M.S.A., Mohammadian T., Hasanzadeh R., Ghasemi M. (2015). Protective and immunogenic effects of *Escherichia* coli-expressed infectious pancreatic necrosis virus (IPNV) VP2-VP3 fusion protein in rainbow trout. Fish Shellfish. Immunol..

[11] Rodriguez S., Alonso M., Perez-Prietol S.I. (2005). Comparison of two birnavirus-rhabdovirus coinfections in fish cell lines. Dis. Aquat. Org..

[12] Panzarin V., Holmes E.C., Abbadi M., Zamperin G., Quartesan R., Milani A., Schivo A., Bille L., Pozza M.D., Monne I. (2018). Low evolutionary rate of infectious pancreatic necrosis virus (IPNV) in Italy is associated with reduced virulence in trout. Virus Evol..

[13] LaPatra S.E., Lauda K.A., Woolley M.J., Armstrong R. (1993). Detection of a naturally occurring co-infection of IHNV and IPNV in rainbow trout. Fish Health Sect. Am. Fish. Soc..

[14] Salonius K., Simard N., Harland R., Ulmer J.B. (2007). The road to licensure of a DNA vaccine. Curr. Opin. Investig. Drugs.

[15] Kouakou K., Schepetkin I.A., Yapi A., Kirpotina L.N., Jutila M.A., Quinn M.T. (2013). Immunomodulatory activity of polysaccharides isolated from *Alchornea cordifolia*. J. Ethnopharmacol..

[16] Ren G., Xu L., Lu T., Yin J. (2018). Structural characterization and antiviral activity of lentinan from *Lentinus edodes* mycelia against infectious hematopoietic necrosis virus. Int. J. Biol. Macromol..

[17] Thuy T.T.T., Ly B.M., Van T.T.T., Quang N.V., Tu H.C., Zheng Y., Seguin-Devaux C., Mi B., Ai U. (2015). Anti-HIV activity of fucoidans from three brown seaweed species. Carbohydr. Polym..

[18] Harden E.A., Falshaw R., Carnachan S.M., Kern E.R., Prichard M.N. (2009). Virucidal activity of polysaccharide extracts from four algal species against herpes simplex virus. Antivir. Res..

[19] Lopes N., Ray S., Espada S.F., Bomfim W.A., Ray B., Faccin-Galhardi L.C., Linhares R.E.C., Nozawa C. (2017). Green seaweed Enteromorpha compressa (*Chlorophyta, Ulvaceae*) derived sulphated polysaccharides inhibit herpes simplex virus. J. Biol. Macromol..

[20] Hidari K.I.P.J., Takahashi N., Arihara M., Nagaoka M., Morita K., Suzuki T. (2008). Structure and anti-dengue virus activity of sulfated polysaccharide from a marine alga. Biochem. Biophys. Res. Commun..

[21] Jin W., Zhang W., Mitra D., McCandless M.G., Sharma P., Tandon R., Zhang F., Linhardt R.J. (2020). The structure-activity relationship of the interactions of SARS-CoV-2 spike glycoproteins with glucuronomannan and sulfated galactofucan from *Saccharina japonica*. Int. J. Biol. Macromol..

[22] Kim M., Yim H.J., Kim S., Kim H.S., Lee W.G., Kim S.J., Kang P., Lee C. (2012). In vitro inhibition of influenza A virus infection by marine microalga-derived sulfated polysaccharide p-KG03. Antivir. Res..

[23] Mendes G.S., Duarte M.E.R., Colodi F.G., Noseda M.D., Ferreira L.G., Berté S.D., Cavalcanti J.F., Santos N., Romanos M.T.V. (2014). Structure and anti-metapneumovirus activity of sulfated galactans from the red seaweed *Cryptonemia seminervis*. Carbohyd. Polym..

[24] Chi Y., Zhang M., Wang X., Fu X., Guan H., Wang P. (2020). Ulvan lyase assisted structural characterization of ulvan from *Ulva pertusa* and its antiviral activity against vesicular stomatitis virus. J. Biol. Macromol..

[25] Chotigeat W., Tongsupa S., Supamataya K., Phongdara A. (2004). Effect of fucoidan on disease resistance of black tiger shrimp. Aquaculture.

[26] Immanuel G., Sivagnanavelmurugan M., Marudhupandi T., Radhakrishnan S., Palavesam A. (2012). The effect of fucoidan from brown seaweed *Sargassum wightii* on WSSV resistance and immune activity in shrimp *Penaeus monodon* (Fab). Fish Shellfish. Immunol..

[27] Ji F., Zhao J., Liu M., Lu T., Liu H., Yin J., Xu L. (2017). Complete genomic sequence of an infectious pancreatic necrosis virus isolated from rainbow trout (*Oncorhynchus mykiss*) in China. Virus Genes.

[28] Zhao J., Xu L., Ren G., Dong Y., Cao Y., Shao Y., Liu H., Yin J., Lu T. (2020). Complete genome sequence and phylogenetic analysis of Sn1203 strain of infectious hematopoietic necrosis virus. Chin. J. Fish..

[29] Miller G.L. (2003). A microtiter modification of the anthrone-sulfuric acid colorimetric assay for glucose-based carbohydrates. Anal. Biochem..

[30] Reed L.J., Münch H. (1938). A simple method of estimating fifty percent endpoints. Am. J. Epidemiol..

[31] Ren G., Xu L., Zhao J., Shao Y., Chen X., Lu T., Zhang Q. (2022). Supplementation of dietary crude lentinan improves the intestinal microbiota and immune barrier in rainbow trout (*Oncorhynchus mykiss*) infected by infectious hematopoietic necrosis virus. Front. Immunol..

[32] Grove J., Marsh M. (2011). The cell biology of receptor-mediated virus entry. J. Cell Biol..

[33] Béress A., Wassermann O., Bruhn T., Béress L., Kraiselburd E.N., Gonzalez L.V., de Motta G.E., Chavez P.I. (1993). A new procedure for the isolation of anti-HIV compounds (polysaccharides and polyphenols) from the marine alga *Fucus vesiculosus*. J. Nat. Prod..

[34] Nguyen T.L., Chen J., Hu Y.L., Wang D., Fan Y., Wang J., Abula S., Zhang J., Qin T., Chen X. (2012). In vitro antiviral activity of sulfated *Auricularia auricula* polysaccharides. Carbohydr. Polym..

[35] Chen Y., Maguire T., Hileman R.E., Fromm J.R., Esko J.D., Linhardt R.J., Marks R.M. (1997). Dengue virus infectivity depends on envelope protein binding to target cell heparan sulfate. Nat. Med..

[36] Modis Y., Ogata S., Clements D., Harrison S.C. (2005). Variable surface epitopes in the crystal structure of dengue virus type 3 envelope glycoprotein. J. Virol..

[37] Kato D., Era S., Watanabe I., Arihara M., Sugiura N., Kimata K., Suzuki Y., Morita K., Hidari K.I.P.J., Suzuki T. (2010). Antiviral activity of chondroitin sulphate E targeting dengue virus envelope protein. Antivir. Res..

[38] Hidari K.I.P.J., Abe T., Suzuki T. (2013). Crabohydrate-related inhibitors of dengue virus entry. Viruses.

[39] Damonte E.B., Matulewicz M.C., Cerezo A.S. (2004). Sulfated seaweed polysaccharides as antiviral agents. Curr. Med. Chem..

[40] Shi Q., Wang A., Lu Z., Qin C., Hu J., Yin J. (2017). Overview on the antiviral activities and mechanisms of marine polysaccharides from seaweeds. Carbohydr. Res..

[41] Wang Y., Chen Y., Du H., Yang J., Ming K., Song M., Liu J. (2016). Comparison of the anti-duck hepatitis a virus activities of phosphorylated and sulfated *Astragalus* polysaccharides. Exp. Biol. Med..

[42] Ming K., Chen Y., Shi J., Yang J., Yao F., Du H., Zhang W., Bai J., Liu J., Wang D. (2017). Effects of *Chrysanthemum indicum* polysaccharide and its phosphate on anti-duck hepatitis a virus and alleviating hepatic injury. J. Biol. Macromol..

[43] Levican J., Miranda-Cardenas C., Soto-Rifo R., Aguayo F., Gaggero A., León O. (2017). Infectious pancreatic necrosis virus enters CHSE-214 cells via macropinocytosis. Sci. Rep..

[44] Mercer J., Helenius A. (2009). Virus entry by macropinocytosis. Nat. Cell Biol..

[45] Chen W., Zhu X., Ma J., Zhang M., Wu H. (2019). Structural elucidation of a novel pectin-polysaccharide from the petal of *Saussurea laniceps* and the mechanism of its anti-HBV activity. Carbohydr. Polym..

[46] Faccin-Galhardi L.C., Aimi Yamamoto K., Ray S., Ray B., Linhares R.E.C., Nozawa C. (2012). The in vitro antiviral property of *Aazadirachta indica* polysaccharides for poliovirus. J. Ethnopharmacol..

[47] Liu C., Chen J., Li E., Fan Q., Wang D., Zhang C., Li P., Li X., Chen X., Qiu S. (2015). Solomonseal polysaccharide and sulfated *Codonopsis pilosula* polysaccharide synergistically resist Newcastle disease virus. PLoS ONE.

[48] Yoo D.G., Kim M.C., Park M.K., Park K., Quan F., Song J., Wee J.J., Wang B., Cho Y., Compans R.W. (2012). Protective effect of Ginseng polysaccharides on influenza viral infection. PLoS ONE.

[49] He X., Fang J., Guo Q., Wang M., Li Y., Meng Y., Huang L. (2020). Advances in antiviral polysaccharides derived from edible and medicinal plants and mushrooms. Carbohydr. Polym..

[50] Xu L., Zhao J., Ren G., Dong Y., Lin J., Cao Y., Yin J., Liu H., Lu T., Zhang Q. (2019). Co-infection of infectious hematopoietic necrosis virus (IHNV) and infectious pancreatic necrosis virus (IPNV) caused high mortality in farmed rainbow trout (*Oncorhynchus mykiss*) in China. Aquaculture.

[51] Lin Q., Fu X., Li N., Wan Q., Chen W., Huang Y., Huang Z., Li J., Zhao L., Lin L. (2017). Co-infections of infectious spleen and kidney necrosis virus and Siniperca chuatsi rhabdovirus in Chinese perch (*Siniperca chuatsi*). Microb. Pathog..

[52] Long A., Garver K.A., Jones S.R.M. (2019). Synergistic osmoregulatory dysfunction during salmon lice (*Lepeophtheirus salmonis*) and infectious hematopoietic necrosis virus co-infection in sockeye salmon (*Oncorhynchus nerka*) smolts. J. Fish Dis..

