# Supplementary material for: Antiviral Activity of Crude Polysaccharide Derived from Seaweed against IHNV and IPNV In Vitro
Source: Viruses. 2022 Sep 19;14(9):2080. doi: 10.3390/v14092080 (PMC9501831; doi:10.3390/v14092080)
Supplement: Supplementary file 1 [file viruses-14-02080-s001.zip › viruses-1898964-supplementary.pdf]

Table S1. Primer sequences for RT-qPCR analysis.

Primers	Forward/Reverse (5' to 3')	Accession Number
IHNV-L	F:TGGGAGCCATTGGTGATT<br>R: GGTTGAGCGTCGGTTTGC	MT242597.1
IPNV-VP2	F:GCATTCAACTACGGGAGAC<br>R: CATCAGGCTGTTGTAGGTTAG	KT274810.1
β-actin	F: CTGTTGGCTTTGGGGTTGAG<br>R: CAGGGAGTGATGGTTGGGATG	AF254414.1

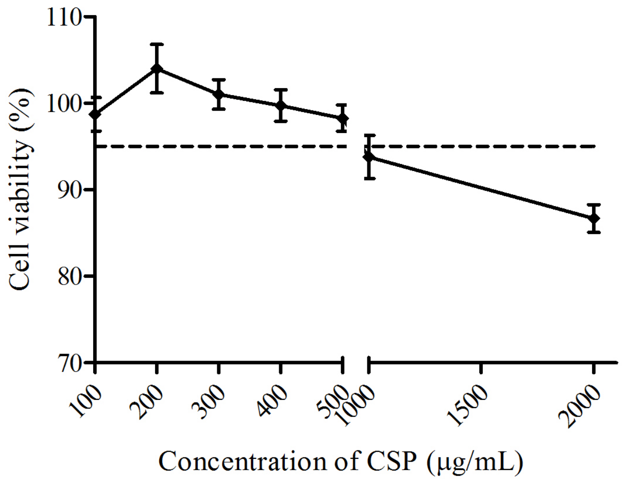

Figure S1. Cytotoxicity analysis of CSP to CHSE-214 cells.
